# Speech comprehension in noisy environments: Evidence from the predictability effects on the N400 and LPC

**DOI:** 10.3389/fpsyg.2023.1105346

**Published:** 2023-02-15

**Authors:** Cheng-Hung Hsin, Pei-Chun Chao, Chia-Ying Lee

**Affiliations:** ^1^Taiwan International Graduate Program in Interdisciplinary Neuroscience, National Yang Ming Chiao Tung University and Academia Sinica, Taipei, Taiwan; ^2^Brain and Language Laboratory, Institute of Linguistics, Academia Sinica, Taipei, Taiwan; ^3^Biomedical Acoustic Signal Processing Lab, Research Center for Information Technology Innovation, Academia Sinica, Taipei, Taiwan; ^4^Institute of Cognitive Neuroscience, National Central University, Taoyuan, Taiwan; ^5^Research Center for Mind, Brain, and Learning, National Chengchi University, Taipei, Taiwan

**Keywords:** predictive processing, predictability effect, speech comprehension, noise, event-related potentials, N400, LPC

## Abstract

**Introduction:**

Speech comprehension involves context-based lexical predictions for efficient semantic integration. This study investigated how noise affects the predictability effect on event-related potentials (ERPs) such as the N400 and late positive component (LPC) in speech comprehension.

**Methods:**

Twenty-seven listeners were asked to comprehend sentences in clear and noisy conditions (hereinafter referred to as “clear speech” and “noisy speech,” respectively) that ended with a high-or low-predictability word during electroencephalogram (EEG) recordings.

**Results:**

The study results regarding clear speech showed the predictability effect on the N400, wherein low-predictability words elicited a larger N400 amplitude than did high-predictability words in the centroparietal and frontocentral regions. Noisy speech showed a reduced and delayed predictability effect on the N400 in the centroparietal regions. Additionally, noisy speech showed a predictability effect on the LPC in the centroparietal regions.

**Discussion:**

These findings suggest that listeners achieve comprehension outcomes through different neural mechanisms according to listening conditions. Noisy speech may be comprehended with a second-pass process that possibly functions to recover the phonological form of degraded speech through phonetic reanalysis or repair, thus compensating for decreased predictive efficiency.

## 1. Introduction

Our innate ability to predict upcoming words based on contextual information is integral to language comprehension. A word’s contextual predictability in a sentence can influence its lexical and semantic processing. For example, readers showed faster reaction times to a predictable word in a supportive context than to an unpredictable word in a neutral context with regard to behavioral tasks, such as lexical decision and word naming (West and Stanovich, 1978; Fischler and Bloom, 1979). Eye-tracking studies have also shown that readers are less likely to fixate on predictable words than on unpredictable words (Ehrlich and Rayner, 1981; Morris, 1994). Moreover, event-related potential (ERP) studies have demonstrated an attenuated N400 amplitude for predictable versus unpredictable words in sentence comprehension (Kutas and Hillyard, 1980, 1984). Research suggests that the predictability effect reflects contextual benefits to comprehension because top-down predictions allow semantic information to be pre-activated to ease lexical access or integration, as opposed to bottom-up input (Kutas and Federmeier, 2011; Kuperberg and Jaeger, 2016). Studies on reading have provided many insights into the predictability effect on the N400 in sentence comprehension tasks. However, only a few ERP studies have investigated predictive processing during speech comprehension, especially under degraded conditions.

The predictability effect during sentence comprehension has been rigorously studied using the N400, a well-established component of the ERPs for semantic processing (Kutas and Federmeier, 2000, 2011). The N400 is a negative-going brain wave that begins around 200–300 ms and peaks around 400 ms after stimulus onset. It is typically distributed in the centroparietal regions of the scalp for reading tasks; for listening tasks, the N400 starts earlier and lasts longer with a more anterior scalp distribution (Kutas and Van Petten, 1994; Van Petten and Luka, 2006). The N400 is most notable for its sensitivity to the cloze probability (operationalized as *predictability*) of a sentence-final word, which can be defined as the proportion of participants who provide a particular word that is most likely to complete a given sentence frame (Taylor, 1953). The N400 amplitude is inversely correlated with the cloze probability of a final word (Kutas and Federmeier, 2000). The difference wave between predictable and unpredictable conditions can readily show the predictability effect on the N400 during sentence comprehension.

Studies have shown that comprehension effortfulness could modulate the predictability effect on the N400 during sentence comprehension. For example, Chang et al. (2016) examined the predictability effect on the N400 during sentence comprehension in people with aphasia, age-matched older adults, and young adults. In a sentence reading task, they found that relative to the young adults, the older adults showed a reduced and delayed predictability effect on the N400 in the posterior regions. The patients with a low comprehension ability showed a frontally distributed N400 with respect to the predictability effect. The anteriority of the predictability effect was found to be negatively correlated to reading proficiency. It has been argued that patients with low reading ability might rely on different mechanisms for making predictions because brain lesions rendered their comprehension effortful (see also Wilson et al., 2012; Tzeng et al., 2018).

The predictability effect could also manifest on the late positive component (LPC), which begins around 600 ms and peaks around 800 ms after stimulus onset in reading tasks; in listening tasks, it peaks later and lasts longer. The LPC exhibits a frontal or parietal scalp distribution depending on whether an unpredicted final word is semantically congruent to the sentence frame (see a review of existing studies by Van Petten and Luka, 2012). An increasing number of studies suggest that the predictability effect on the LPC during sentence comprehension is associated with second-pass processing attempts for semantic integration (DeLong et al., 2014; Brothers et al., 2020; DeLong and Kutas, 2020; Kuperberg et al., 2020). These studies demonstrated that sentences with semantic anomalies or disconfirmed predictions could induce the predictability effect on the LPC because the reader is required to undergo second-pass efforts through reanalysis or revision to make sense of the input. Studies of aging also show the LPC to follow a reduced and delayed N400 with regard to the predictability effect, suggesting that older readers may employ different comprehension strategies as owing to their age, they are less reliant on contextual information than young readers (DeLong et al., 2012; Chang et al., 2016; Xu et al., 2017; Dave et al., 2018; Zhu et al., 2018).

Studies on speech comprehension also demonstrated the effects of predictability and semantic congruency on the N400 and LPC, suggesting that listeners may use contextual information to predict upcoming linguistic inputs (McCallum et al., 1984; Holcomb and Neville, 1991; Van Petten et al., 1999; Hagoort and Brown, 2000; van den Brink and Hagoort, 2004; Boudewyn et al., 2015). However, only a few N400 studies have examined how degraded speech affects the use of context during listening comprehension (Connolly et al., 1992; Aydelott et al., 2006; Obleser and Kotz, 2011; Strauß et al., 2013; Coulter et al., 2021; Silcox and Payne, 2021). A typical finding across these studies is that the amplitude and latency of the N400 regarding the predictability effect were reduced and delayed as a function of decreased speech clarity. For example, Obleser and Kotz (2011) manipulated the cloze probability of the sentence-final word (high vs. low) under three levels of acoustic degradation. They found that the predictability effect on the N400 decreased linearly with increasing degradation, suggesting that poor speech quality limited the availability of contextual information for efficient lexical and semantic information processing. Strauß et al. (2013) further manipulated contextual constraint (strong vs. weak) and the typicality of the sentence-final word (high vs. low) under three levels of acoustic degradation. Likewise, their results showed parametric reductions of the predictability effect on the N400 as acoustic degradation increased. Thus, Strauß et al. (2013) proposed the *Expectancy Searchlight Model* to explain how speech quality affects predictive processing during comprehension. For clear speech, listeners use contextual cues to actively predict (i.e., the *searchlight*) a list of plausible lexical candidates for the upcoming words. For degraded speech, since auditory and lexical analyses depend on shared cognitive capacities, effortful processing of speech signals captures resources that would be used for predictive processing to operate normally. Therefore, the searchlight narrows the range of lexical items to only the most probable candidates, reducing the predictability effect on the N400.

A question that remains unclear is whether speech clarity affects the predictability effect on the LPC. Boudewyn et al. (2015) manipulated global predictability and local feature consistency to investigate the use of the global and local contexts for upcoming words during speech comprehension. They showed graded effects of global predictability and local consistency on the N400, suggesting that upcoming words compatible with context-based predictability and feature consistency would receive a processing benefit as the N400 amplitudes were reduced. Moreover, their results showed interactive effects between global predictability and local consistency on the post-N400 positivity (PNP), indicating increased demands on revision or updating processes relating to semantic P600 and LPC effects in other studies (Van Petten and Luka, 2012; DeLong et al., 2014; Brothers et al., 2020; DeLong and Kutas, 2020; Kuperberg et al., 2020). The PNP effect suggests that unpredictable but plausible input may have listeners abandon their expectations and update their representations accordingly. However, no studies have reported the predictability effect on the LPC in speech comprehension under adverse listening conditions. The only exception might be Daltrozzo et al. (2012), who manipulated the semantic congruency of the sentence-final word and noise levels. The congruency effect was evident on the N400 and LPC in clear speech but was reduced and absent under mild and substantial noise. These findings suggest that speech clarity affects reanalysis attempts for semantic integration. Nevertheless, it remains unknown whether the predictability effect on the LPC would manifest in noise for unpredictable but semantically congruent sentences.

This study thus aimed to examine whether and how speech clarity affects the predictability effect on the N400 and LPC during speech comprehension. Participants were instructed to listen to sentences that ended with a high-or low-predictability word in clear and noisy conditions. We expected to replicate the typical predictability effect on the N400 in the clear condition. According to the *Expectancy Searchlight Model*, the noisy condition was expected to show a reduced predictability effect on the N400, followed by the LPC. Furthermore, the topographic distribution of the predictability effect on the N400 and LPC, namely if they were distributed frontally or posteriorly, may shed some light on how listeners allocate cognitive resources for speech comprehension in noisy conditions.

## 2. Materials and methods

### 2.1. Participants

Twenty-seven healthy young adults participated in this study (six males, age = 20–30 years, mean = 23.48, *SD* = 2.76). All participants were right-handed, well-educated (mean years of education = 16.69, *SD* = 1.22), and native speakers of Mandarin Chinese with normal or corrected-to-normal vision. None reported a history of neurological or psychiatric disorders or brain damage. Each participant was tested for hearing acuity at 500, 1,000, 2,000, and 4,000 Hz using pure-tone audiometry (PTA). All participants showed normal hearing with a hearing threshold below 25 dB in their better hearing ear. All recruitment and experimental procedures, including informed consent and data privacy, complied with the ethical conduct for human research regulated by the local Institutional Review Board (IRB) at Academia Sinica.

### 2.2. Stimuli

This study manipulated the predictability of the sentence-final word (high vs. low) and speech clarity (clear vs. noisy). The stimuli included 128 declarative sentences in Mandarin Chinese, 75 of which were adapted from Chang et al. (2016). The sentences contained 9–16 characters and were all semantically plausible and syntactically simple (Table 1). Each sentence was embedded with a disyllabic final word. Half of the total number of final words were strongly constrained by the sentence frame and highly predictable (mean cloze probability = 0.83, *SD* = 0.14, range 0.48–1). The remaining words were weakly constrained by the sentence frame and less predictable (mean cloze probability = 0.02, *SD* = 0.04, range 0–0.21). All final words were selected from Academia Sinica Balanced Corpus of Modern Chinese (Huang and Chen, 1998) and matched for word frequency (*F_1, 124_* = 0.01, *p* = 0.91), contextual diversity (*F_1, 124_* = 0.16, *p* = 0.69), semantic diversity (*F_1, 124_* = 0.01, *p* = 0.94), visual complexity (first character: *F_1, 124_* = 1.3, *p* = 0.26; second character: *F_1, 124_* = 1.63, *p* = 0.2), and orthographic neighborhood size (*F_1, 124_* = 0.85, *p* = 0.36).

**Table 1:** Example sentences of high-and low-predictability conditions.

Condition	Predictability	Example Sentences							
High predictability	0.83 (0.14)	千萬 Absolutely	不要 do not	出賣 sell	自己的 self		靈魂 soul	給… to…	**魔鬼 devil**
		(Never sell your soul to the devil.)							
Low predictability	0.02 (0.04)	他 He	計畫 plans	下個 next	月 month	要 to	前往… go to…	**沙漠 desert**	
		(He plans to go to the desert next month.)							

Target words are in bold in the example sentences. Values in the predictability column are the mean cloze probability and standard deviation (in parentheses) of the sentence-final word.

Regarding the auditory stimuli, the 128 sentences were generated as clear or noisy speech using Audacity (version 2.1.3). The stimuli were recorded at 16 bits with a sampling rate of 44.1 kHz by a female native speaker of Mandarin Chinese and were natural in speed, intonation, and prosody (mean length of sentences = 4.1 s, *SD* = 0.61; mean length of target words = 0.78 s, *SD* = 0.1). To create the noisy condition, half of the sentences were masked by random snippets of news reports that simulated indoor acoustics for comprehension of noisy speech. Each sentence was masked by a snippet that starts and ends with the sentence onset and offset. Snippets by female anchors were excluded to avoid voice confusion. The masking was created with a signal-to-noise ratio (SNR) at +8 dB. This SNR was employed based on the standardized Speech Perception in Noise (SPIN) test (Kalikow et al., 1977; Bilger et al., 1984) and field research on ambient noises (Pearsons et al., 1977; Olsen, 1998). The stimuli were normalized and presented at a 65 dB sound pressure level (SPL), that is, approximately the level of daily conversation.

### 2.3. Procedure

Each participant was seated individually approximately 120 cm in front of a projection screen in a sound-attenuated and electrically shielded room. The experiment was presented *via* Psychtoolbox 3 in MATLAB environment (R2014b). All auditory stimuli were presented binaurally through a set of two loudspeakers placed 90 cm in front of the participant. Before EEG recording, each participant was reminded to refrain from producing muscle and ocular artifacts to the extent possible during each stimulus presentation. Subsequently, the participants received 12 practice trials to familiarize themselves with the experimental procedure. During practice, the researchers of the study ensured that each participant could hear and read the stimuli clearly. A sound level meter controlled the maximum intensity level at a 65 dB SPL. The formal recording session included 128 randomized trials delivered in four blocks. Each block lasted approximately 7–8 min, and every participant was free to take a break between blocks for as long as they needed.

During each trial, a fixation cross appeared at the center of the screen for 500 ms to prepare the participant for an incoming stimulus. Subsequently, with the fixation, an auditory stimulus (i.e., a spoken sentence) was presented, and each stimulus appeared only once. Each participant was instructed to attend to the stimulus for listening comprehension (2,500–5,000 ms). Following the end of each stimulus, the fixation cross remained onscreen for another 2,000 ms. Subsequently, the computer showed a yes-no comprehension question or a forced word-choice question on the screen regarding the sentence that had just been presented. For example, after the participant had heard *Never sell your soul to the devil*, the computer showed *Do you sell your soul to the devil?—yes/no* or *Which word did you hear?—devil/wire*. The participant responded by pressing buttons that represented *yes/no* or a word choice on a keyboard. The questions were counterbalanced and the participant’s behavioral accuracies were recorded. This task was used to ensure that the participants were attentive during the stimulus presentation. Two types of questions were introduced to ensure that the participants attended not only to the sentence-final word but also to the overall meaning of the sentences. To minimize EEG artifacts, each participant was instructed not to respond until the question appeared. However, the experiment proceeded only after the question was answered.

### 2.4. EEG/ERP acquisition

Electroencephalogram/ERPs were recorded from 64 sintered Ag/AgCl electrodes (QuickCap, Neuromedical Supplies, Sterling, Texas, United States). All electrodes were referenced online to the vertex of the scalp between the Cz and CPz electrodes. A ground electrode was placed anterior to the Fz electrode. The EEG was recorded continuously and digitized at 1,000 Hz. The signals were amplified using SynAmps2 (Neuroscan Inc.) with a 0.05–200 Hz band-pass filter. For further analysis, the data were re-referenced offline to the average of the left and right mastoids (M1 and M2). A pair of electrodes was placed on the supraorbital and infraorbital ridges of the left eye to record vertical eye movements and blinks. Another pair of electrodes was placed on the outer canthi for horizontal eye movements. The impedance of all electrodes was maintained below 5 kΩ.

### 2.5. EEG/ERP pre-processing

The EEG data were re-referenced offline with Compumedics Neuroscan 4.5 software and pre-processed with FieldTrip (Oostenveld et al., 2011). The EEG data were segmented into epochs that were time-locked to the onset of the target word from −100 ms pre-stimulus onset to 1,500 ms post-stimulus onset. The pre-stimulus interval was used for baseline correction. The segmented EEG data were low-pass filtered at 30 Hz across all channels. Eye movement artifacts identified by independent component analysis (ICA) were removed from all segmented trials. Trials with voltage variations larger than 100 μV were rejected. Overall, the trial rejection rate was 6.89%. After pre-processing, the remaining trials were computed using Ensemble Empirical Mode Decomposition (EEMD) analysis (Huang et al., 1998; Wu and Huang, 2009).

### 2.6. Ensemble empirical mode decomposition analysis

The Ensemble Empirical Mode Decomposition (EEMD) is a data-driven approach for handling nonlinear and nonstationary time-frequency analysis (e.g., EEG and ERP data). This adaptive algorithm has been proven to be exceptionally effective in optimizing the signal-to-noise ratio (SNR) for a better estimate of ERP latency and amplitude (Al-Subari et al., 2015; Hsu et al., 2016; for a review of existing studies, see Sweeney-Reed et al., 2018). The present study followed the procedure of EEMD analysis described by Hsu et al. (2016) to generate intrinsic mode functions (IMFs), which represent the local properties of events in terms of time and frequency. When EEMD was computed, Gaussian white noise was added to the signal of each EEG trial of each channel, and the amplitude of the Gaussian noises was set at 10% of the EEG signal’s standard deviation. This algorithm was applied to decompose the noise-assisted signal into eight IMFs and one residual trend. These steps were repeated 10 times, sifting with different white noises to produce 40 ensembles of corresponding IMFs (Hsu et al., 2016; Tzeng et al., 2017; Chao et al., 2019). The resultant IMFs were obtained by averaging all ensembles of each IMF.

Hilbert spectral analysis was employed to evaluate the time-frequency spectra of the IMFs. The results revealed that IMF 5 showed a central frequency at 9.35 Hz, ranging from 0 to 16.33 Hz; IMF 6 showed a central frequency at 4.04 Hz, ranging from 0 to 8.78 Hz; IMF 7 showed a central frequency at 1.98 Hz, ranging from 0 to 4.85 Hz; and IMF 8 showed a central frequency at 1.01 Hz, ranging from 0 to 4.41 Hz. The present study performed a summation across IMF 6, IMF 7, and IMF 8 (Chen et al., 2016; Chao et al., 2019) to cover the frequency range of 0–8.78 Hz, subsequently averaging over all trials for each condition in each channel to yield event-related modes (ERMs).

### 2.7. Statistical analysis

**Figure 1:**
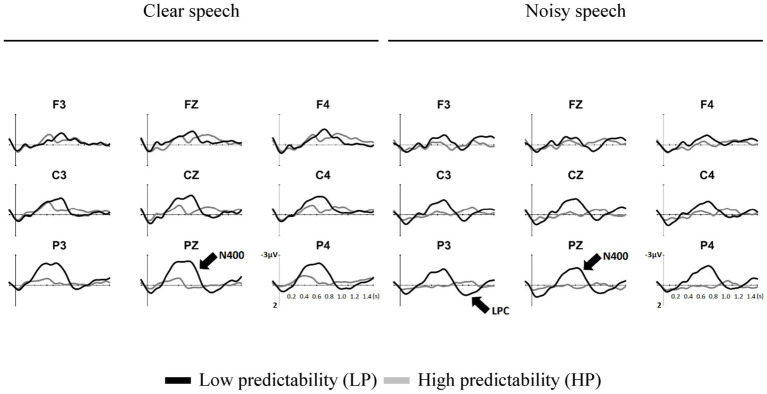
The averaged event-related modes (ERMs) of the low- (black line) and high- (grey line) predictability words in the clear condition (left) and the noisy condition (right).

Figure 1 shows the grand-averaged ERMs elicited by the low-and high-predictability words. Visual inspection of the data showed the N400 from 300 to 900 ms and the LPC from 900 to 1,300 ms with a posterior distribution. The primary aim of this study was to determine whether the clarity of speech revealed different predictability effects on the N400 and LPC during speech comprehension. Therefore, first, cluster-based permutation tests were conducted (Maris and Oostenveld, 2007) to characterize the temporal dynamics and topographic distributions of predictability effects in the two clarity conditions (i.e., clear speech and noisy speech). Subsequently, linear mixed models (LMMs) analysis was performed (Baayen et al., 2008) to examine whether speech clarity would modulate the predictability effect on the N400 and LPC during speech comprehension.

### 2.8. Temporal and spatial cluster-based permutation tests

Cluster-based permutation tests are a nonparametric statistical method that elegantly handles the multiple comparison problem in high-dimensional EEG data (Sassenhagen and Draschkow, 2019). This method is a mass univariate approach that considers the correlated neural activities at adjacent time points and electrodes when estimating effects. This study used FieldTrip (Oostenveld et al., 2011) to perform cluster-based permutation tests to examine the predictability effect on the N400 and LPC for clear speech and noisy speech, respectively. The epochs of 350–850 and 950–1,200 ms of each condition of each electrode were divided into 10 and 5 successive time windows of 50 ms intervals. Cluster-based permutation tests were performed on the mean amplitudes of each time window on each electrode by employing the following steps. First, for the predictability effects in clear speech and noisy speech, a simple dependent-samples *t*-test was performed at each time point and each electrode. The algorithm identified all time points and electrodes that exceeded a certain significance level (α = 0.05) and formed clusters. Temporal or spatial clusters were formed when *t*-statistics values of at least three adjacent time points or electrodes exceeded the abovementioned significance level. A cluster mass was calculated for each cluster by taking the sum of all the individual *t*-statistics within that cluster. Subsequently, the algorithm randomly sorted the event codes of the experimental conditions to create a null distribution of the maximum cluster mass through 1,000 permutations. The algorithm identified clusters at each permutation and found the maximum cluster mass. Cluster correction was used to control the familywise error rate for multiple comparisons. Lastly, the cluster masses that were observed in the data were compared against this null distribution, and only cluster masses in the highest or lowest 2.5th percentile were considered significant.

### 2.9. Linear mixed models analysis

Linear mixed models (Baayen et al., 2008) were used to examine if the speech clarity would modulate the predictability on the N400 and LPC in a fixed time window. This analysis was performed using the lme4 (Bates et al., 2015) package (version 1.1.23) in the R environment (version 4.0.0; R Core Team, 2020). Using single-trial ERP data, the LMMs calculated the differences between the mean amplitudes for the low-and high-predictability sentences in the clear and noisy conditions in each temporal window of interest over each region of interest (ROI). The temporal windows and ROIs were defined based on the results from cluster-based permutation tests on the main effects of predictability. The temporal windows of interest were defined from 400 to 800 ms for the N400, and from 1,050 to 1,150 ms for the LPC. Two ROIs were defined: anterior (Fz, F1, F2, F3, F4, FCz, FC1, FC2, FC3, and FC4) and posterior (Cz, C1, C2, C3, C4, CPz, CP1, CP2, CP3, CP4, Pz, P1, P2, P3, P4, POz, PO3, PO4, PO5, and PO6). This analysis simultaneously models the variance associated with each participant and item as random effects. Therefore, the LMMs were estimated by including predictability (high/low), clarity (clear/noisy), and the interaction between these two effects in the ROIs as fixed effects, as well as the participants and items as crossed random effects. The estimated coefficient (*β*), standard error (*SE*), and *t*-value for fixed effects are listed in Table 2 for the N400 and Table 3 for the LPC.

**Table 2:** LMM estimates of fixed effects for the predictability and clarity effects on the N400.

ROIs	Variables	N400 (400–800 ms)			
		Beta	Std. Error	*t* value	
Anterior	(Intercept)	−0.6316	0.1571	−4.019	^***^
	Predictability	−0.4313	0.1726	−2.499	^*^
	Clarity	−0.1133	0.1676	−0.676	
	Predictability *×* Clarity	−0.3498	0.3462	−1.010	
Posterior	(Intercept)	−0.8145	0.1420	−5.737	^***^
	Predictability	−1.5766	0.1374	−11.475	^***^
	Clarity	−0.0925	0.1313	−0.705	
	Predictability *×* Clarity	−0.9716	0.2758	−3.523	^***^

Predictability: The contrast between low-and high-predictability sentences. Clarity: The contrast between clear and noisy conditions. Predictability *×* Clarity: The interaction effect between Predictability and Clarity. ^*^p < 0.05; ^**^p < 0.01; ^***^p < 0.001.

**Table 3:** LMM estimates of fixed effects for the predictability and clarity effects on the LPC.

ROIs	Variables	LPC (1,050–1,150 ms)			
		Beta	Std. Error	*t* value	
Anterior	(Intercept)	−0.2462	0.2157	−1.142	
	Predictability	1.5963	0.2396	6.661	^***^
	Clarity	−0.1884	0.2316	−0.814	
	Predictability *×* Clarity	−0.8903	0.4808	−1.851	
Posterior	(Intercept)	0.1161	0.1783	0.651	
	Predictability	1.6685	0.1743	9.572	^***^
	Clarity	−0.9201	0.1665	−5.525	^***^
	Predictability *×* Clarity	−0.7568	0.3499	−2.163	^*^

Predictability: The contrast between low-and high-predictability sentences. Clarity: The contrast between clear and noisy conditions. Predictability *×* Clarity: The interaction effect between Predictability and Clarity. ^*^p < 0.05; ^**^p < 0.01; ^***^p < 0.001.

## 3. Results

### 3.1. Behavioral performance

The study results showed that the participants achieved an overall mean accuracy of 99.54% (*SD* = 0.7, range = 97.64–100%). A two-way repeated measures ANOVA was performed to analyze the effects of predictability and speech clarity on behavioral accuracy. The results showed no significant interactions between the effects of predictability and speech clarity (*F_1, 104_* = 0.0002, *p* > 0.05). The simple main effects analysis revealed a significant predictability effect on accuracy (*F_1, 104_* = 5.66, *p* < 0.05), wherein the accuracy was significantly higher for the low-predictability sentences (99.59% ± 1.06, *mean ± SD*) than the high-predictability ones (99.48% ± 1.46, *mean ± SD*). However, the analysis showed no significant effects of speech clarity on accuracy (*F_1, 104_* = 0.24, *p* > 0.05).

### 3.2. Cluster-based permutation tests

**Figure 2:**
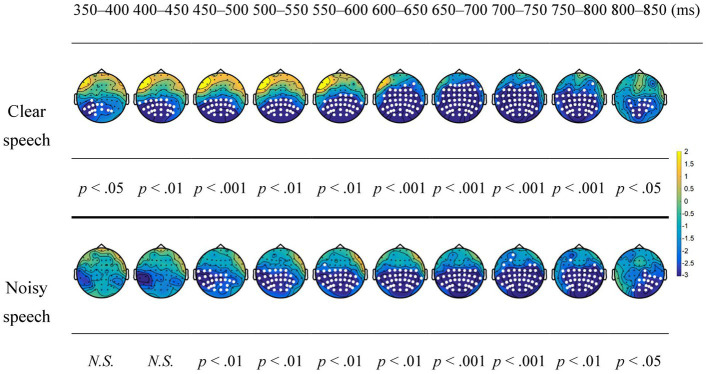
Topographic maps for the predictability effect on the N400 under the clear and noisy conditions in a moving time window analysis. White dots represent the electrodes that show significant differences in the contrasts. N.S., not significant.

Figure 2 shows the results of cluster-based permutation tests for the predictability effect (low–high) under the clear and noisy conditions on the N400 in a moving time window analysis. Under the clear condition, the predictability effect showed a significant negative cluster in the centroparietal and frontocentral regions from 350 to 850 ms (*p*s < 0.05). Under the noisy condition, the predictability effect showed a significant negative cluster mainly in the centroparietal regions from 450 to 850 ms (*p*s < 0.05). Compared to clear speech, the predictability effect on the N400 showed a delayed latency and a less anteriorly spread scalp distribution in noisy speech.

**Figure 3:**
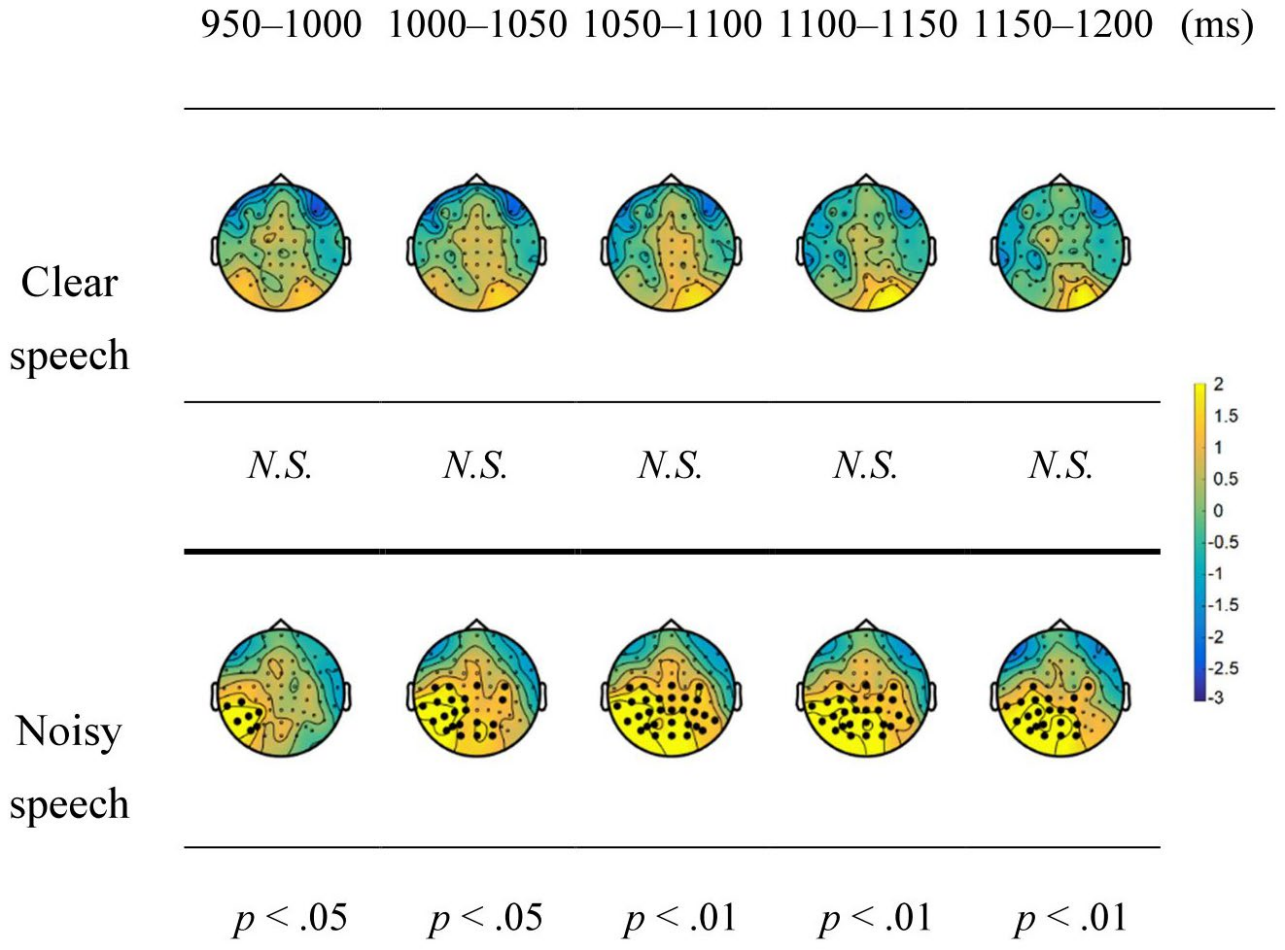
Topographic maps for the predictability effect on the late positive component (LPC) under the clear and noisy conditions in a moving time window analysis. Black dots represent the electrodes that show significant differences in the contrasts. N.S., not significant.

Figure 3 shows the results of cluster-based permutation tests for the predictability effect under the clear and noisy conditions on the LPC in a moving time window analysis. Under the clear condition, the predictability effect showed no significant clusters from 950 to 1,200 ms, whereas under the noisy condition, the predictability effect showed a significant positive cluster in the centroparietal regions during 950–1,200 ms (*p*s < 0.05).

### 3.3. LMM analysis

#### 3.3.1. N400 (400–800 ms)

Table 2 summarizes the results of LMM analysis of the predictability, clarity, and interaction between these two effects on the N400s in the anterior and posterior ROIs. The LMM analysis showed a significant interaction effect between predictability and clarity in the posterior ROI (*β* = −0.9716, *SE* = 0.2758, *p* < 0.001) but not in the anterior ROI (*β* = −0.3498, *SE* = 0.3462, *p* > 0.05). *Post hoc* analysis showed that the predictability effect under the noisy condition (*β* = −1.091, *SE* = 0.198, *z ratio* = −5.512, *p* < 0.0001) was smaller than under the clear condition (*β* = −2.062, *SE* = 0.191, *z ratio* = −10.776, *p* < 0.0001). The low-predictability sentences yielded greater negative N400s than the high-predictability sentences. Additionally, the LMM analysis revealed significant main effects of predictability on the N400 in both anterior (*β* = −0.4313, *SE* = 0.1726, *p* < 0.05) and posterior ROIs (*β* = −1.5766, *SE* = 0.1374, *p* < 0.001), wherein the low-predictability sentences elicited greater negativity of the N400 than the high-predictability sentences. However, the LMM analysis showed no main effects of clarity on the N400 in both anterior (*β* = −0.1133, *SE* = 0.1676, *p* > 0.05) and posterior ROIs (*β* = −0.0925, *SE* = 0.1313, *p* > 0.05).

#### 3.3.2. LPC (1,050–1,150 ms)

Table 3 summarizes the results of LMM analysis of the predictability, clarity, and interaction between these two effects on the LPCs in the anterior and posterior ROIs. The LMM analysis showed a significant interaction effect between predictability and clarity in the posterior ROI (*β* = −0.7568, *SE* = 0.3499, *p* < 0.05), but not in the anterior ROI (*β* = −0.8903, *SE* = 0.4808, *p* > 0.05). *Post hoc* analysis showed that the predictability effect under the noisy condition (*β* = 2.047, *SE* = 0.251, *z ratio* = 8.153, *p* < 0.0001) was larger than under the clear condition (*β* = 1.290, *SE* = 0.243, *z ratio* = 5.313, *p* < 0.0001). The low-predictability sentences yielded greater positive LPCs than the high-predictability sentences. Additionally, the LMM analysis revealed significant main effects of predictability on the LPC in both anterior (*β* = 1.5963 *SE* = 0.2396, *p* < 0.001) and posterior ROIs (*β* = 1.6685, *SE* = 0.1743, *p* < 0.001), wherein the low-predictability sentences elicited greater positivity of the LPC than the high-predictability sentences. Lastly, the LMM analysis revealed a significant main effect of clarity on the LPC in the posterior ROI (*β* = −0.9201, *SE* = 0.1665, *p* < 0.001); further, it was observed that noisy speech elicited greater positivity of the LPC than clear speech. The LMM analysis showed no significant main effects of clarity on the LPC in the anterior ROI (*β* = −0.1884, *SE* = 0.2316, *p* > 0.05).

## 4. Discussion

This study investigated how noise affects predictive processing in speech comprehension. The behavioral results showed a ceiling effect on speech comprehension regardless of clarity, suggesting that the participants were attentive to performing the task. This ceiling effect also indicated well-preserved comprehension of the participants at the behavioral level. Moreover, a predictability effect on the N400 was demonstrated for clear speech in the centroparietal and frontocentral regions. However, noisy speech exerted a predictability effect mainly in the centroparietal regions. Considering results pertaining to ERPs, noisy speech showed a delayed and significantly reduced predictability effect on the N400 compared to clear speech, as demonstrated by cluster-based permutation tests and the LMM analysis. Critically, in noisy speech, the N400 for the predictability effect was followed by a posterior LPC. Overall, the study results suggest that speech comprehension relies on a second-pass process when noise compromises predictive processing.

### 4.1. The predictability effect on the N400 during speech comprehension

The study results regarding clear speech demonstrated a predictability effect on the N400, which is in line with previous findings (Holcomb and Neville, 1991; van den Brink and Hagoort, 2004; Boudewyn et al., 2015). Of note, in connected speech, contextual benefits can attenuate N400 waveforms to a large extent that the N400 becomes less visually identifiable (Woodward et al., 1993; Besson et al., 1997). In previous studies, it has been proposed that the predictability effect on the N400 reflects the relative difficulty of lexical integration (Connolly and Phillips, 1994; Van Petten et al., 1999; van den Brink and Hagoort, 2004). Nevertheless, recent studies view the predictability effect on the N400 as active predictions of upcoming input facilitated by a supportive context (Boudewyn et al., 2015). Regarding noisy speech, the predictability effect was reduced and delayed on the N400. It was suggested that degraded speech signals restricted the availability of contextual information for predictive processing to operate efficiently (Aydelott et al., 2006; Obleser and Kotz, 2011; Strauß et al., 2013; Silcox and Payne, 2021). Strauß et al. (2013) asked listeners to comprehend clear and degraded sentences of high and low contextual constraints and typicality. Their results showed graded predictability effects on the N400 in the clear condition, but not in the degraded conditions. The moderately predictable sentences did not show contextual benefits for comprehension in the degraded conditions. Moreover, their data demonstrated a delayed predictability effect on the N400 when the speech was moderately degraded, as compared to the clear speech. Based on the *Expectancy Searchlight Model*, the reduced and delayed predictability effect on the N400 in the degraded conditions indicates that the listeners could not predict upcoming words effectively because they could not utilize sentential context to make liberal predictions of lexical candidates. In line with this model, this study’s results for the predictability effect on the N400 suggest that speech comprehension in noisy conditions becomes effortful because context-based lexical and semantic predictions cannot be carried out effectively.

Nevertheless, this study’s participants achieved significantly high accuracy for comprehension at the behavioral level regardless of speech clarity level. This is in line with previous studies that found high behavioral accuracies and subjective ratings for their comprehension under degraded conditions in various tasks. Silcox and Payne (2021) found that the well-preserved comprehension of noisy speech at the behavioral level seemed to come at the cost of poorer memory recall of the speech content. However, it is still unclear how listeners achieve well-preserved comprehension outcomes under adverse listening conditions. Chang et al. (2016) showed an anteriorly shifted predictability effect on the N400 in aphasic patients with a low comprehension ability. Based on a functional neuroanatomic model for semantic processing of words in context (Lau et al., 2008), the study suggested that a frontally distributed predictability effect on the N400 may reflect controlled processes for retrieving and selecting lexical representations among other competing representations. However, this study did not find a frontally distributed predictability effect on the N400 in the noisy condition. It appears that in healthy adults, moderate noise does not cause language processing to be effortful to the extent that it demands other mechanisms to engage for comprehension (see also Silcox and Payne, 2021). For low-ability patients, reading comprehension is probably a far more effortful task. Clinical and developmental studies have reported that the topographic changes of the N400 correlated with proficiency in language use (Wilson et al., 2012; Tzeng et al., 2017, 2018). In Chang et al. (2016), patients with a high comprehension ability showed a posteriorly distributed predictability effect on the N400. It appears that the severity of language impairment may drive the predictability effect on the N400 to shift anteriorly. The low-ability patients seem to readily require additional and immediate support from other mechanisms in the first-pass processing to retrieve lexical and semantic representations correctly. However, speech comprehension in noisy conditions seems to rely on different mechanisms for successful comprehension outcomes.

### 4.2. The predictability effect on the LPC for noisy speech

In this study, the noisy condition revealed the predictability effect on the LPC in the posterior regions. Van Petten and Luka (2012) demonstrated that the LPC for predictive processing can be categorized into a frontal LPC and posterior LPC with distinctive functional roles. The frontal LPC is often observed when an unexpected but plausible word disconfirms predictions in a highly constraining context, whereas the posterior LPC is commonly observed for a semantically anomalous word regardless of contextual constraint (DeLong et al., 2014; Quante et al., 2018; Brothers et al., 2020; DeLong and Kutas, 2020; Kuperberg et al., 2020). While the frontal LPC was attributed to increased efforts to integrate an unexpected but plausible word into the context, the posterior LPC was interpreted as integration difficulties of an incongruent word that caused semantic anomalies. It appears that unexpectedness and implausibility are the defining features of a word to elicit the frontal and posterior LPCs.

Current studies suggest that the frontal LPC reflects the inhibition of the incorrectly predicted information so that the unexpected but plausible word that actually appears could be integrated into the context (Kutas, 1993; DeLong et al., 2014; Ness and Meltzer-Asscher, 2018; DeLong and Kutas, 2020). An alternative view is that the frontal LPC reflects a representation updating process for the new unpredicted information (Brothers et al., 2015, 2020; Kuperberg et al., 2020). Concerning the posterior LPC, the idea of event structure has been proposed to account for semantic anomalies (Kuperberg and Jaeger, 2016; Brothers et al., 2020; Kuperberg et al., 2020). The event structure can be seen as an extended concept of thematic roles, underscoring the predictable agent-patient relation in a broader context (e.g., discourse). For example, *The lifeguards received a report of sharks right near the beach. Their immediate concern was to prevent any incidents in the sea. Hence, they cautioned the swimmers/drawer* (Kuperberg et al., 2020). The predictability effect on the posterior LPC was observed because *drawer* is thematically implausible in this context; thus, the event structure of this discourse is violated. Therefore, second-pass attempts are made through reanalysis or repair for new interpretations, as reflected by the posterior LPC.

Although the LPC as a component reflects a modality-general process (Van Petten and Luka, 2012), only a few studies have investigated how the LPC manifests for the predictability or congruency effect under adverse listening conditions. Daltrozzo et al. (2012) examined how different noise levels affect the congruency effect in speech comprehension. They found that clear speech elicited the congruency effect on the N400 and LPC in the central and posterior regions. Moreover, the congruency effect on the N400 and LPC was reduced and delayed as a function of decreased speech clarity, suggesting that the congruency effect on the posterior LPC seemed to reflect a repair process for word meaning identification. However, the significance of the graded effects of congruency on the LPC is unclear. A parsimonious explanation is that this reflects an increasingly compromised repair process under increasing noise. Alternatively, following the *Expectancy Searchlight Model*, it can be interpreted that there is less information to be repaired because fewer predictions were made under the increasing noise.

Ryskin et al. (2021) suggested that the predictability effect on the posterior LPC may reflect a recovery process for noise-corrupted inputs during reading comprehension. They used highly constraining sentences and manipulated the syntactic and semantic congruency of the final word based on orthographical similarity in a reading task. For example, *The storyteller could turn any incident into an amusing anecdote/anecdotes/hearse/antidote.* They showed the predictability effect on the N400, but not on the LPC, between the congruent and semantically incongruent conditions (i.e., *anecdote* vs. *hearse*). The absence of the LPC suggested that *hearse* was an error because *hearse* did not share any similarities with *anecdote* in that particular context. The context provides a low probability for *hearse* to be considered a corrupted form of *anecdote* for recovery. Moreover, they showed the predictability effect on the posterior LPC, but not on the N400, between the congruent and the syntactically incongruent conditions (i.e., *anecdote* vs. *anecdotes*). The context provides a high probability for *anecdotes* to be accepted as a corrupted form of *anecdote* for recovery because these two words are nearly identical, differing only in inflection. Lastly, they showed a reduced N400 amplitude followed by a posterior LPC for the predictability effect between the congruent and orthographically similar conditions (i.e., *anecdote* vs. *antidote*). In this case, the context provides a relatively high probability for a*ntidote* to be accepted as a corrupted form of *anecdote* because these two words are orthographically similar. Critically, they showed that the N400 and LPC amplitudes were positively correlated with the accuracy of word recovery. Ryskin et al. (2021) argued that a reader would estimate the likelihood of the intended sentence given the perceived form in noise. A corrupted input could be recovered by inferring its correct form through its orthographical or phonological neighbors within a context. Therefore, the predictability effect on the posterior LPC may reflect a recovery process of word form.

In the present study, the clear condition did not show any LPCs for the predictability effect because the sentences chosen as stimuli were all plausible without violations of strong predictions. Neither semantic nor perceptual factors caused anomalies for second-pass attempts. However, the noisy condition showed a reduced N400 followed by a posterior LPC for the predictability effect, in line with Ryskin et al. (2021). This study’s results suggest that speech comprehension likely engages a similar recovery process of word form when ambient noise causes phonetic anomalies. Specifically, speech comprehension in noise involves a second-pass process that possibly functions to recover the phonological forms of degraded speech through phonetic reanalysis or repair. Indeed, such a mechanism could be especially important when comprehension occurs under moderate noise, as in this study. Notably, this study’s participants achieved well-preserved comprehension outcomes by using different underlying neural mechanisms according to the listening conditions, supporting a word form recovery process. Nonetheless, the recovery process in this study may operate differently from that in Ryskin et al. (2021). In their study, the recovery process depended on a specific reference word that was made available through lexical predictions in a highly constraining context. However, this study used neutral contexts to form the low-predictability sentences. No words could be made specific as the reference. Therefore, the listener made second-pass attempts to recover the word form through phonetic reanalysis or repair based on the perceived form in noise and its close alternatives in the mental lexicon. This phonetic reanalysis or repair process likely occurs to consolidate representations when the listener can perceive a coarse word form under moderate noise. The listener may be unable to reanalyze speech sounds through second-pass efforts when overwhelming noise allows little phonetic information to be perceived. The present findings on the LPC may explain how listeners in previous studies maintained intact behavioral performance while showing reduced predictability effects on the N400 under some degraded conditions (e.g., Aydelott et al., 2006; Boulenger et al., 2011). In addition, these results resonate with those from studies on cognitive aging that showed well-preserved reading and speech comprehension at the behavioral level but reduced N400 effects and enhanced frontal LPC effects in older adults relative to young adults, suggesting that other neural mechanisms may be used to compensate for language comprehension (Federmeier et al., 2003, 2010; Faustmann et al., 2007; DeLong et al., 2012; Dave et al., 2018, 2021).

## 5. Conclusion

This study provides novel evidence to demonstrate the LPC as listeners’ second-pass attempts to achieve comprehension outcomes through phonetic reanalysis when ambient noise compromises predictive processing. While the ERP literature has primarily focused on reading for meaning-level semantic anomalies, the present findings show the LPC when form-level phonetic and phonological anomalies occur in the auditory modality. The word form recovery process seems to reflect humans’ behavioral tendency to recheck what has been said and heard during a loud conversation. This study suggests that the LPC is a versatile component that responds to cognitive, linguistic, and perceptual factors in distinctive ways for language comprehension. Additionally, this study showed that news reports with a moderate SNR were sufficiently noisy to compromise the online processing of lexical and semantic information in young adults. The informational content of news reports created a semantic masking effect (Presacco et al., 2016). Since noise with linguistic information is virtually ubiquitous, this study may provide some ecological validity with respect to indoor acoustics for speech comprehension in everyday life. Thus, this study’s results show that it takes more than a shallow message-level representation to comprehend noisy speech (cf. Silcox and Payne, 2021). Listeners make second-pass attempts to mitigate the impact of decreased predictive efficiency in noise through phonetic reanalysis or repair of word forms.

## Data availability statement

The raw data supporting the conclusions of this article will be made available by the authors, without undue reservation.

## Ethics statement

The studies involving human participants were reviewed and approved by IRB on Biomedical Science Research, Academia Sinica. The patients/participants provided their written informed consent to participate in this study.

## Author contributions

C-HH conceived the study, designed the experiment, collected and analyzed the data, interpreted the results, and wrote the manuscript. P-CC analyzed the data and revised the manuscript. C-YL conceived the study, designed the experiment, analyzed the data, interpreted the results, and revised and proofread the manuscript. All authors contributed to the article and approved the submitted version.

## Funding

This work was supported by the Institute of Linguistics, Academia Sinica.

## Conflict of interest

The authors declare that the research was conducted in the absence of any commercial or financial relationships that could be construed as a potential conflict of interest.

## Publisher’s note

All claims expressed in this article are solely those of the authors and do not necessarily represent those of their affiliated organizations, or those of the publisher, the editors and the reviewers. Any product that may be evaluated in this article, or claim that may be made by its manufacturer, is not guaranteed or endorsed by the publisher.
